# Methods Used in Smoking Cessation and Reduction Attempts: Findings from Help-Seeking Smokers

**DOI:** 10.1155/2021/6670628

**Published:** 2021-03-09

**Authors:** Marianne Lund, Elisabeth Kvaavik

**Affiliations:** Norwegian Institute of Public Health, Oslo, Norway

## Abstract

In addition to traditional smoking cessation methods like nicotine replacement therapy (NRT), new methods such as mobile applications and e-cigarettes have been added to the toolbox. The purpose of this study was to examine which methods smokers currently use in quit or reduction attempts and map characteristics of users of the various methods. In this study, participants were smokers who visited a website or called a quit line for smoking cessation and who were currently in quit or reduction attempts (*N* = 740). Data were collected in Norway in 2013–2017 through a web survey. Most smokers were currently trying to quit, and the most frequently used methods were a smoking cessation app for mobile phones, nicotine replacement therapies (NRTs), and e-cigarettes. Logistic regression analyses identified older daily smokers with high cigarette consumption as NRT users, while the users of a cessation app were younger females. The use of e-cigarettes was associated with older, low educated smokers with low cigarette consumption. The use of the mobile phone app was associated with having made several recent quit attempts. The study provides insight into help-seeking smokers' preferences for smoking cessation methods and user characteristics. This knowledge is relevant for further work in smoking cessation planning and policies.

## 1. Introduction

Smoking cessation in the adult population is essential to accelerate the reduction in smoking-related morbidity and mortality. In Norway, nearly one in ten adults aged between 16 and 79 years were daily smokers in 2019 and a similar proportion was occasional smokers [1]. Smoking prevalence in the youth population (13–15 years) was below 3% in 2019 [2], indicating positive prospects for decreasing smoking-related deaths. On the European tobacco control scale, Norway ranks as number five, but with a low score on tobacco treatment [3]. As many as 75% of smokers in Norway have an intention to quit, which indicates a potential for increased quitting activity [4]. Increasing successful smoking cessation in the adult population will improve population health substantially, but empirical evidence shows that many smokers struggle to quit.

In general, unassisted quitting is the most common route to smoking cessation, although studies indicate that some form of evidence-based smoking cessation aid is better than no aid, and multiple methods may increase the chances of successfully quitting compared to the use of a single method [5–7]. Several methods or cessation aids are available for smokers who want to quit, including nicotine replacement therapy (NRT), prescription medication (bupropion/varenicline), behavioral counseling and quit lines, apps, and websites for smoking cessation. Financial incentives have also been used to increase quit rates, improving smoking cessation [8]. In Norway, NRT has been available for over-the-counter sales since 2003, and an intervention study with free smoking cessation medication in addition to standard cessation counseling for heavy smokers is ongoing.

In the last decade, e-cigarette use has increased and e-cigarettes have become an additional smoking cessation aid [9, 10]. In Norway, the highest share of e-cigarette users is found among former and daily smokers. Reported reasons for use are better health and stigma from smoking combustible cigarettes [1, 11]. E-liquid with nicotine is not presently allowed to be sold in Norway, but the ban is expected to be lifted in 2021, in line with the EU's Tobacco Product Directive. In 2019, the prevalence of daily snus use (moist oral tobacco) was 13% in Norway and use was concentrated among young males. Daily cigarette smoking was 9%, with the highest rate among people 50 years or older. The availability of an alternative tobacco product possibly plays a role in smoking cessation in Norway and Sweden [12, 13]. The use of NRT, prescription medication like varenicline, and e-cigarettes increases success rates in smoking cessation interventions and randomized control trials (RCT) [14–16]. Also, combining several NRT products results in higher long-term quit rates than using a single NRT product [17].

An increase in the use of assistance for smoking cessation has been reported previously, as well as increased internet searches for information about smoking cessation aids [7, 18]. However, smokers' preferences for smoking cessation methods are gradually changing over time, with increasing quit attempts without any assistance and higher use of e-cigarettes [19].

Smokers seeking assistance for smoking cessation are more likely to be women, older smokers, and heavy smokers [7]. A recent study found that younger smokers, in addition to heavier smokers and those with previous quit attempts, were more willing than older smokers to use an evidence-based smoking cessation method [20]. Smokers with high consumption of cigarettes have a stronger preference for choosing pharmacotherapy than do light smokers [7, 20, 21].

Quit success rates, duration of use, reduced side effects, and price are important factors for smoker's preferences for choice of cessation methods [22, 23]. In an experimental study, the likelihood of quit success and reduced side effects were valued as an important feature in the choice of smoking cessation medication, and smokers were willing to pay a higher price for cessation medication with high efficacy [23].

Reasons for using e-cigarettes among smokers are reported as follows: to allow the choice of different flavors, to reduce regular smoking, to save money, and to cause less harm to health than ordinary cigarettes [10]. An online survey investigating preferences for smoking cessation methods reported the highest interest in NRT, websites with quitting advice, and prescription medication [21]. This study also revealed that smokers were least interested in smoking cessation methods involving interpersonal interaction and rather preferred nonsocial cessation methods, such as the internet and pharmacotherapy [21].

Most studies investigating preferred cessation methods normally include just a few alternatives, often excluding e-cigarettes. The aim of the present study is to explore a variety of smoking cessation methods used by smokers who plan to quit or reduce smoking, including the use of e-cigarettes. We investigate user characteristics for each smoking cessation method separately; sociodemographic characteristics, smoking behavior characteristics, and quitting plan (reduce or quit). Since the combination of different types of cessation methods is considered more effective, we aim to investigate factors related to the use of multiple cessation methods as compared to the use of one single method.

## 2. Materials and Methods

Callers to a quit line (“Røyketelefonen”) and users of a website for tobacco use cessation (http://www.slutta.no, hereafter called “slutta.no”), both run by the Norwegian Directorate of Health, were invited to participate in a study about quitting or reducing tobacco consumption, including cigarettes and/or snus. The present analytical sample includes current smokers with a plan to quit smoking or reduce their cigarette consumption. Current smokers without a quitting plan, former smokers, exclusive snus users, or those only seeking information on tobacco use were excluded from the analytical sample. Callers or website users below 16 years of age were excluded from participation in the study.

Those who agreed to participate gave their email address, either to the quit line operator or via a website link to the study. Recruitment from one source (quit line or website) excluded study participation from the other source. The market research company Norfakta Markedsanalyse AS administered the distribution of the online survey and the data collection. Participation was voluntary, and participants could at any time withdraw their consent and have their data deleted. The research protocol was submitted for consideration to the Regional Committee for Medical and Health Research Ethics, where the project was considered to be beyond the scope of the Health Regulation Act. A notification was made to the Norwegian Social Science Data Service, due to the processing of personal data (no. 35567). Those who completed the survey at each round joined a draw of 10 gift cards for NOK 1 500 each, corresponding to 140 € or 167 US$.

### 2.1. Outcome Measures

Daily and occasional smokers with a current plan to either quit or reduce smoking were asked about their current use of smoking cessation methods. A total of 16 potential cessation methods were listed, with the response options “yes” and “no” in respect of the current use of the listed cessation method. The respondents could register multiple cessation methods. The respondents recruited from the website slutta.no were not given slutta.no as an alternative option in the question about smoking cessation methods. Those recruited from the quit line were given the option of quit line calling due to an additional question regarding the possibility of call back telephone counseling. To gain an equal treatment of the group, we did not include quit line calling in the analysis of smoking cessation methods. The recruitment method indicates that all participants in the study have used at least one smoking cessation method.

The wording of smoking cessation methods was as follows: “In your current attempt to quit or reduce your smoking, do you use some of the following methods?” Methods belonging to the same category were merged. The use of NRT consisted of four items (nicotine gum, patch, lozenge, and inhalator), and those who answered “yes” to at least one item were defined as current users of NRT. Prescription medication was defined as the use of at least one of two medications (Zyban (bupropion) and/or Champix (varenicline)). The use of e-cigarettes constituted one single question, without specifying whether the respondent used e-cigarettes with or without nicotine. The use of snus was covered by one item. The use of smoking cessation applications (apps) consisted of two items, one related to the specific use of the *slutta* app, developed by the Norwegian Directorate of Health, the other concerning “other cessation apps” in general. Since 232 participants reported having used the *slutta* app specifically and 39 reported the use of other apps, this item mainly refers to the use of the *slutta* app. One item asked about the use of internet sites delivering smoking cessation aids other than the one they were recruited from (“slutta.no”). We also included one question relating to the use of social media platforms such as Facebook, Twitter, and Instagram for smoking cessation aids. One question asked about attendance at smoking cessation courses, but very few reported this option, and the item was therefore omitted in the analysis. The questions are listed in Supplementary Materials, Appendix table (available here).

### 2.2. Independent Variables

Sociodemographic variables were gender, age, and education level. Participants were grouped into three age groups: 16–29 years, 30–49 years, and 50 years or above. Educational level was originally measured on six levels: seven years of primary school, nine years of primary and lower secondary school, one to two years of upper secondary school, the third year of upper secondary school, one to five years of higher education (e.g., bachelor degree), and four or more years of higher education (master's degree or higher). Educational level was grouped for analysis into low educational level (with upper secondary education as the highest level) and high educational level (lower and higher university level).

Daily and occasional smokers were asked about consumption of cigarettes per day and cigarettes per week, respectively. To obtain one common measure for cigarette consumption, cigarettes per day were converted to cigarettes per week for daily smokers. Previous quit attempts measured the number of quit attempts in the last 12 months, categorized into no quit attempts, one quit attempt, and two or more.

The variable multiple methods were constructed by counting all the dichotomous variables of single methods (NRT, e-cigarettes, app, snus, medication, social media, and other internet sites), giving a variable ranging from 0 to 6. This was recoded into 0 (=no additional method used, i.e., in addition to the use of the website or quit line), 1 (=one additional method used), 2 (=two additional methods used), and 3 (=three or more additional methods used) and for the logistic regression model recoded into a new variable of single versus multiple methods used.

### 2.3. Statistical Analysis

Differences between the sociodemographic variables, plans, cigarette consumption, and quit attempts and the outcome variable smoking cessation methods were analyzed using logistic regression analysis. The regression models were computed as follows: model 1, sociodemographic variables only; model 2, sociodemographics+smoking status; model 3, sociodemographics+cigarette consumption; model 4, sociodemographics+plans; and model 5, sociodemographics+previous quit attempts. Results are presented in Table 1 as adjusted odds ratios (aOR) with 95% confidence intervals.

Logistic regression analysis was also used to investigate the association between sociodemographic and smoking behavior characteristics and the use of multiple methods (two or more), compared to a single method. This analysis included only those who reported the use of any of the listed methods (*N* = 590).

## 3. Results

The total sample enrolled 2,517 participants aged 16 years or older in 2013–2017. Most participants were recruited from “slutta.no” (90%); see Supplementary Materials, Table 5. Our analytical sample consists of smokers who stated that they currently planned to quit smoking or reduce their cigarette consumption (*N* = 740). Descriptive statistics are presented in Table 2.

The total sample did not reflect the population in respect of gender, age, and educational level, with an overrepresentation of females, age group 30–49 years, and higher educated individuals, see Supplementary Materials, Table 5. Former smokers constituted the majority of the total sample (56%), while about one-third reported current smoking. Eighteen percent were daily snus users. The analytical sample (current smokers) had the same distribution for the demographic variables as the total sample, but fewer used snus in the analytical sample than in the total sample.

### 3.1. Sociodemographic Characteristics

Among cigarette smokers currently in a quitting or reduction process (*N* = 740), more smokers were planning to quit their smoking (83%) than reduce their cigarette consumption (17%) (Table 2). The majority were daily smokers (84%) and about 9% were dual users of cigarettes (daily and nondaily combined) and snus (daily use only), 58% smoked more than 70 cigarettes per week, and a majority (86%) had tried to quit in the last year. One-quarter of the sample (25%) did not use any of the listed smoking cessation methods. The most common methods used were e-cigarettes (26%), NRTs (26%), and cessation apps (37%). Snus used as a cessation method was reported by seven percent, indicating that approximately half of dual users of cigarettes and snus use snus as a means of quitting.

Older smokers were significantly more likely to have used NRT, e-cigarettes, and cessation medication in their current attempt to quit or reduce smoking (Table 1). The use of snus and smoking cessation apps was more likely in the youngest age group. Gender differences were observed, with a higher odds ratio for females to use the cessation apps and a higher odds ratio for men to use snus. Educational differences were only observed for the use of e-cigarettes, which was more likely among smokers with short education compared to those with long education.

### 3.2. Smoking Behavior Characteristics

The use of NRT was associated with daily smoking and high cigarette consumption, while the use of smoking cessation medication was only significantly associated with high weekly cigarette consumption. E-cigarette use was associated with occasional smoking and low cigarette consumption. No significant association was observed between the use of snus, other websites, and smoking behavior. The use of social media as a smoking cessation aid was associated with low cigarette consumption, but not with smoking status.

### 3.3. Multiple Methods versus One Single Method

Those who planned to quit as opposed to reduce their smoking had higher odds ratios for using multiple smoking cessation methods compared to using only one single method (Table 3). The cessation methods most often used in combination with other methods was the cessation app (64%). NRT was mentioned by half of those who used multiple cessation methods and e-cigarettes by 38% (data not shown).

## 4. Discussion

The most frequent method used, the smoking cessation app developed by the Norwegian Directorate of Health, was most prevalent among the young, women, and those with a plan to quit. In general, females are more likely to use smoking cessation aids than men [24], but the association between gender and mobile apps for cessation is unclear. A recent Dutch study found no association between sociodemographic variables and intention to use a mobile app for smoking cessation [25].

Previous research on gender differences in smoking cessation medication use (both NRT and prescription medication) reports higher use among females [26]. In our sample, we did not observe gender differences in NRT or medication use, but a higher odds ratio was observed for snus use as a cessation method among males. A previous study of a representative sample of Norwegian smokers found that snus use was the most commonly reported cessation method used by males, while NRT was the most common cessation method used by females [27].

The finding that smokers with lower educational level are more likely to use e-cigarettes as a smoking cessation method is of interest. There is a strong need for quitting methods which enable increasing quitting activity among vulnerable groups or smokers with low educational level, to overcome social inequalities in smoking-related morbidity and mortality. Systematic review studies on this topic do not support the suggestion that e-cigarettes may reduce smoking inequality [28, 29].

Age was the sociodemographic variable that most clearly characterized the users of the various smoking cessation methods. There seems to be a generation gap between the use of NRT, e-cigarettes, and prescription medication on the one hand and smoking cessation apps and snus use on the other. There is some support in the literature for older smokers preferring NRT, e-cigarettes, and smoking cessation medication [30–32]. However, a study from 27 EU Member States found that younger smokers were more likely to have used e-cigarettes for smoking cessation [19].

The observed association between the number of quit attempts and the use of a cessation app is supported by others [25, 33]. The constant reminding and stimulation in a quitting process that the cessation apps provide may stimulate quit attempts, although the causal direction is not known. Further investigation of the preferences for using cessation apps and of the reasons for the association between quit attempts and the use of a mobile app is warranted.

Established smokers may prefer a smoking cessation method that handles both abstinence and nicotine dependence and replace an established habit of cigarette smoking with the activity of vaping [34, 35]. This assumption is supported by our findings that daily smokers and those with high cigarette consumption per week have higher odds of using NRT and prescription medication. The lack of an association between high-consuming cigarette smokers and e-cigarettes may be explained by the fact that nicotine-containing e-liquid is not permitted to be sold in Norway or that e-cigarette users are more likely to reduce their consumption and make a switch in their smoking status from daily to occasional smoking.

There are several smoking cessation apps on the market, but their effectiveness for successful smoking cessation is inconclusive [36]. Some single studies have found effects [37–39]. Their potential as a tool in smoking cessation is considered to be high, with low cost, high reach, and a “choice architecture” potential, i.e., they are capable of influencing individual decision-making in social environments where choices need to be made [40]. The nudge approach to health behavior change highlights “choice architecture,” individual autonomy, and simplicity in behavioral change [41, 42]. Studies on smoking cessation applications are constantly evolving, and the use of smart technology such as the possibility of detecting smoking in real time may be an effective tool for increasing success rates [43, 44].

NRTs have been on the market for a long time, with a variety of available product types. They are easily accessible, easy to use, and considered safe for most adults, even for long-term use [45]. The Norwegian guidelines on smoking cessation suggest minimal intervention, including advice to use pharmacotherapy (NRT or prescription medication). The sale of nicotine replacement products, both in pharmacies and over the counter, increased from 2015 to 2019 [46]. The effect of NRTs in smoking cessation is considered high in treatment settings, particularly among heavy smokers, but their real-world effectiveness is disputable [47–49].

Smokers' choices of cessation methods seem to reflect the recommendations made by the health authorities and health personnel regarding NRTs and the smoking cessation app, but not e-cigarettes. The Norwegian tobacco policy on e-cigarettes as a smoking cessation method is in line with the WHO's approach, and the recommendation for e-cigarette use in smoking cessation has been categorized as “precautionary nonuse” [50]. The Norwegian government's tobacco control strategy does not dismiss the idea of a harm reduction perspective [51]. However, e-cigarettes are not recommended as a smoking cessation method in the national guidelines on smoking cessation [52]. In that light, the number of smokers reporting e-cigarettes as a smoking cessation method may be seen as high.

The prevalence of e-cigarette use in Norway is low, and use is most prevalent among current and former smokers [1]. The evidence for e-cigarettes' role in smoking cessation is increasing and may explain the high use of e-cigarettes as a smoking cessation method in our sample of help-seeking smokers [15, 19, 53]. Another explanation for the relatively high use of e-cigarettes in our study may be related to an incident at the start of the study, where the owner of a vaping website promoted the present study to its members. This promotion was removed on our recommendation and would therefore have influenced the first part of the data collection only. This incident may have led to artificially high numbers of smokers using e-cigarettes as a cessation method in the study.

Although e-cigarettes containing nicotine are not yet on the market in Norway and e-cigarettes are not included in the governmental toolbox of smoking cessation methods, information about e-cigarettes is highly visible in a variety of media channels, from user organizations, vape shops, webshops, and by word of mouth [54, 55]. The availability of e-cigarettes, including nicotine e-liquid at cross-border shopping sites and online, and its affordability may explain the relatively high use among smokers planning to quit.

Few respondents in this sample used snus as a smoking cessation method. This finding stands in contrast to previous findings on former male smokers in Norway, where snus was the most used method to quit [27]. One possible explanation is selection bias. Due to the self-recruitment strategy, the sample is not representative of Norwegian smokers.

## 5. Limitations

A limitation of this study is that the findings may not apply to the general population of smokers in Norway (see Supplementary Materials, Table 5). The study sample is a convenient sample of help-seeking smokers. Although the recruitment was from two different platforms, very few were recruited from the quit line. One possible explanation is the difference in total visits, with 190 000 visitors on the website in 2014, while less than 10 000 called the quit line in the same year. Those who were invited from the quit line were also given the opportunity of a postal survey, although very few opted for this solution. The majority were recruited from the website, and recruitment was thereby restricted to those who were able to complete an online survey.

Receiving advice from health care professionals is often included as an important part of the cessation aid toolbox, but that was not addressed in the present study.

## 6. Conclusions

The majority of the study participants in the current study used NRTs, a smoking cessation app, or e-cigarettes as methods for smoking cessation. Older smokers were more likely to use NRTs and e-cigarettes, while younger smokers were more likely to use the smoking cessation app. Females were more likely to use the cessation app, and males more likely to use snus. E-cigarette use was more common among smokers with low educational level. This knowledge is relevant for further work in smoking cessation planning and policies.

## Figures and Tables

**Table 1:** Logistic regression models of the association between sociodemographic and smoking behavior characteristics and current use of cessation methods. Adjusted odds ratios (aOR) and 95% confidence intervals.

	Current use of NRT *N* = 190 aOR (95% CI)	Current use of e-cigarettes *N* = 192 aOR (95% CI)	Current use of cessation app *N* = 271 aOR (95% CI)	Current use of snus *N* = 55 aOR (95% CI)	Current use of cessation medication *N* = 75 aOR (95% CI)	Current use of social media *N* = 55 aOR (95% CI)	Current use of other websites *N* = 47 aOR (95% CI)
*Model 1*							
Age 30–49 vs. 16–29	1.75 (1.02-3.02)	2.44 (1.40-4.24)	0.52 (0.34-0.80)	0.88 (0.45-1.73)	2.47 (0.95-6.45)	0.96 (0.44-2.11)	1.26 (0.50-3.17)
Age 50+ vs. 16–29	2.26 (1.29-3.97)	2.08 (1.16-3.73)	0.25 (0.15-0.39)	0.21 (0.08-0.57)	3.20 (1.21-8.49)	0.97 (0.42-2.23)	1.27 (0.48-3.34)
Females vs. males	1.33 (0.91-1.95)	0.77 (0.54-1.11)	1.55 (1.08-2.19)	0.53 (0.30-0.94)	1.48 (0.83-2.64)	1.09 (0.59-2.02)	1.14 (0.58-2.26)
High vs. low education	1.08 (0.77-1.52)	0.62 (0.44-0.86)	1.25 (0.91-1.71)	0.99 (0.56-1.74)	1.34 (0.81-2.20)	0.97 (0.56-1.69)	1.44 (0.78-2.67)
*Model 1+smoking status*							
Daily vs. occasional smoking	2.11 (1.20-3.72)	0.35 (0.22-0.54)	1.04 (0.68-1.61)	0.61 (0.31-1.20)	1.84 (0.77-4.41)	0.71 (0.35-1.46)	1.95 (0.67-5.66)
*Model 1+CPW*							
21–70 per week vs. 20 or less per week	1.86 (0.96-3.58)	0.32 (0.19-0.56)	0.87 (0.51-1.47)	0.62 (0.26-1.49)	1.21 (0.37-1.89)	0.25 (0.10-0.61)	1.27 (0.39-4.15)
71 per week or more	2.30 (1.25-4.26)	0.41 (0.26-0.67)	1.32 (0.82-2.14)	0.60 (0.28-1.29)	3.07 (1.07-8.81)	0.50 (0.25-1.01)	1.77 (0.60-5.25)
*Model 1+plans*							
Plan to quit vs. plan to reduce	1.48 (0.91-2.41)	1.45 (0.90-2.33)	2.68 (1.66-4.32)	0.66 (0.34-1.29)	1.65 (0.76-3.54)	1.05 (0.50-2.21)	2.22 (0.78-6.32)
*Model 1+quit attempts*							
1 vs. none	0.96 (0.56-1.64)	1.76 (1.01-3.06)	1.44 (0.86-2.43)	1.52 (0.56-4.14)	2.00 (0.86-4.64)	1.48 (0.55-4.00)	0.93 (0.36-2.42)
2 or more vs. none	1.42 (0.84-2.41)	1.36 (0.77-2.39)	1.92 (1.14-3.22)	1.42 (0.52-3.88)	1.34 (0.56-3.20)	1.76 (0.65-4.69)	1.24 (0.49-3.16)

**Table 2:** Descriptive statistics of smokers planning to quit smoking or reduce their cigarette consumption recruited from quit line and web page for smoking cessation, 2013–2017 (*N* = 740).

	Analytical sample 16–81 years (*N* = 740)	
	*N*	%
Gender		
Male	213	28.7
Female	527	71.3
Age group (years)		
16–29	118	16.0
30–49	379	51.3
50–81	242	32.8
Education		
Low level	332	44.9
High level	408	55.1
Smoking status		
Daily	624	84.3
Occasional	116	15.7
Former	—	—
Never	—	—
Former/never		
Snus use status		
Daily	65	8.8
Occasional	61	8.2
Former	104	14.1
Never	510	68.9
Recruited from		
Website	639	86.4
Quit line	101	13.7
Current plan		
Reduce smoking	126	17.0
Quit smoking	614	83.0
No plan	—	—
Refuse to answer	—	—
Cigarettes per week (CPW)		
<20	100	13.9
21–70	204	28.3
71+	416	57.8
Previous quit attempts last 12 months		
0	100	13.5
1	319	43.2
2+	320	43.3
Number of cessation methods used in addition to quit line/website^1^		
0	187	25.3
1	325	43.9
2	158	21.4
3+	70	9.5
NRT (4 items)	190	26.0
E-cigarettes (1 item)	192	26.0
Cessation app (2 items)	271	36.6
Snus (1 item)	55	7.4
Cessation medication (2 items)	75	10.1
Social media (1 item)	55	7.4
Other cessation websites	47	6.4

^1^0 refers to those who answered no use of any of the listed smoking cessation methods.

**Table 3:** Logistic regression analysis for multiple use of cessation methods among smokers who reported the use of at least one additional method (*N* = 590). Crude and adjusted odds ratio (aOR).

	Multiple versus single methods used	Multiple versus single methods used
	Crude OR	aOR (95% CI)
Age		
Age 30–49 vs. 16–29	1.53 (0.93-2.51)	1.50 (0.88-2.55)
Age 50+ vs. 16–29	1.28 (0.75-2.20)	1.37 (0.77-2.44)
Gender		
Females vs. males	1.21 (0.83-1.77)	1.14 (0.77-1.70)
Education		
High vs. low education	1.09 (0.77-1.53)	1.09 (0.76-1.55)
Smoking status		
Daily vs. occasional smoking	0.99 (0.63-1.55)	1.09 (0.51-2.32)
Cigarettes per week (CPW)		
21–70 CPW vs. 20 or less CPW	0.63 (0.35-1.10)	0.54 (0.24-1.19)
71 or more CPW vs. 20 or less CPW	0.98 (0.60-1.60)	0.83 (0.38-1.80)
Plan to quit or reduce		
Plan to quit vs. plan to reduce	1.82 (1.07-3.10)	1.75 (1.01-3.04)
Quit attempts last 12 months		
1 vs. none	1.32 (0.75-2.41)	1.24 (0.68-2.27)
2 or more vs. none	1.51 (0.84-2.70)	1.51 (0.82-2.78)

## Data Availability

Data are available upon reasonable request.

## Supplementary Materials

Supplementary Materials Supplementary Materials are added: Appendix table, an overview of the listed smoking cessation methods, and Table 5, an overview over the total sample, including population numbers (link).
