# Supplementary material for: Methods Used in Smoking Cessation and Reduction Attempts: Findings from Help-Seeking Smokers
Source: J Smok Cessat. 2021 Mar 9;2021:6670628. doi: 10.1155/2021/6670628 (PMC8279185; doi:10.1155/2021/6670628)
Supplement: Supplementary Materials — are added: Appendix table, an overview of the listed smoking cessation methods, and Table 5, an overview over the total sample, including population numbers (link). [file 6670628.f1.docx]

Supplementary Materials.

Appendix table. Variable list:

“Do you use some of these methods in your current attempt to quit or reduce smoking?”

(Yes/No)

Nicotine gum

Nicotine patch

Nicotine lozenge

Nicotine inhalator

E-cigarettes

Quit-line (you called)

Quit-line (follow-up call)

Website slutta.no (only given to callers to the quitline)

Other web-sites (other than [www.slutta.no](http://www.slutta.no))

The mobile app slutta

Other smoking cessation apps

Social media (like Facebook and such)

Snus

Smoking cessation courses

Zyban

Champix

None of the above

Table 5: Study sample and population data.

|  | Total sample  16-81 years  (N=2 517) | | Norwegian population^1^  16-81 years  (N=3 885 030) | |
| --- | --- | --- | --- | --- |
|  | N | % | N | % |
| Gender |  |  |  |  |
| Male | 950 | 37.7 | 1 967 283 | 50.6 |
| Female | 1 567 | 62.3 | 1 917 747 | 49.4 |
| Age group (years) |  |  |  |  |
| 16-29 | 536 | 21.3 | 932 146 | 24.0 |
| 30-49 | 1 279 | 50.8 | 1 416 806 | 36.5 |
| 50 -81 | 703 | 27.9 | 1 536 078 | 39.5 |
| Education |  |  |  |  |
| Low level | 1 128 | 44.9 | 2 753 721 | 69.6 |
| High level | 1 389 | 55.1 | 1 203 824 | 30.4 |
| Smoking status |  |  |  |  |
| Daily | 713 | 28.3 | [571 099] | 14.7 |
| Occasional | 146 | 5.8 | [330 228] | 8.5 |
| Former | 1 404 | 55.8 |  |  |
| Never | 254 | 10.1 |  |  |
| Non (former/never) |  |  | [2 983 703] | 76.8 |
| Snus use status |  |  |  |  |
| Daily | 446 | 17.7 | [341 883] | 8.8 |
| Occasional | 133 | 5.3 | [159 286] | 4.1 |
| Former | 580 | 23.0 |  |  |
| Never | 1 362 | 54.0 |  |  |
| Non (former/never) |  |  | [3 383 861] | 87.1 |
| Recruited from |  |  |  |  |
| Website | 2 262 | 89.9 |  |  |
| Quit Line | 255 | 10.1 |  |  |
| Current plan |  |  |  |  |
| Reduce smoking | 126 | 14.7 |  |  |
| Quit smoking | 614 | 71.5 |  |  |
| No plan | 109 | 12.7 |  |  |
| Refuse to answer | 10 | 1.2 |  |  |

^1^2013-numbers
